# Recent Advances in Understanding, Diagnosing, and Treating Ovarian Cancer

**DOI:** 10.12688/f1000research.9977.1

**Published:** 2017-01-27

**Authors:** Kathryn Mills, Katherine Fuh

**Affiliations:** 1 Washington University School of Medicine, St. Louis, MO, USA

**Keywords:** Ovarian cancer, cancer mechanisms, gene mutation, diagnosis and prevention

## Abstract

Ovarian cancer, a term that encompasses ovarian, fallopian, and peritoneal cancers, is the leading cause of gynecologic cancer mortality. To improve patient outcomes, the field is currently focused on defining the mechanisms of cancer formation and spread, early diagnosis and prevention, and developing novel therapeutic options. This review summarizes recent advances in these areas.

## Introduction

Ovarian cancer was first identified in 1959 by Dr Martin Swerdlow, who described a malignant pelvic mass that surrounded the left fallopian tube but did not involve the mucosal epithelium. This tumor was thought to develop from tissue with an origin similar to that of the ovary, such as the pelvic peritoneum, fallopian tubes, or uterus ^1^. Since then, cancers of these tissues have been collectively referred to as Müllerian adenocarcinomas to reflect the fact that we often do not know exactly which organ these tumors originate from. However, for simplicity, we will refer to these tumors by their more common name, ovarian cancers. Ovarian cancer is the most lethal gynecologic malignancy and is the fifth most common cause of cancer death in women ^2, 3^.

The majority of women with ovarian cancer are diagnosed with advanced-stage disease; only 15% of all cases are diagnosed with local disease ^2, 3^. Since the 1970s, the five-year survival for all stages has improved from 30% to 46% ^3^ as a result of taxane and platinum chemotherapies, intraperitoneal (IP) administration of chemotherapy, and risk-reduction surgeries. However, five-year survival for advanced disease, such as stage IIIC, is a mere 39%. Risk factors for ovarian cancer include family history, nulliparity, lack of breast feeding, and infertility ^4^. In addition, between 5% and 15% of all women with ovarian cancer have inherited mutations in DNA repair genes such as *BRCA1*, *BRCA2*, and genes associated with Lynch syndrome ^4– 7^. *BRCA1*, *BRCA2*, and Lynch mutations increase the lifetime risks of ovarian cancer by as much as 60-, 30-, and 13-fold, respectively ^5, 8^. Currently, the field is focused on defining the mechanisms of cancer formation and spread, early diagnosis and prevention, and developing novel therapeutic options. This review highlights some of the newest findings in these areas.

## Mechanisms of ovarian cancer formation and spread

### Tumor types

The majority of ovarian cancers are of epithelial histology, and high-grade serous carcinomas (HGSCs) are the most common, but there are other epithelial histologic subtypes, such as clear cell, endometrioid, and mucinous ^4^. These can be divided into two major subtypes: type I and type II. Type I cancers (clear cell, endometrioid, and low-grade serous) grow slowly and seem to develop in a step-wise process. For example, low-grade serous tumors arise from benign serous cystadenoma or Müllerian inclusion cysts that accumulate mutations in pathways such as KRAS and BRAF ^9^. Likewise, clear cell and endometrioid carcinomas may originate from endometriosis as shown by the finding that the prevalence of self-reported endometriosis was higher in women with clear cell (20%) and endometrioid (14%) cancers than in those with low-grade serous (9.2%) or mucinous (6%) cancers ^10^. Type II tumors are characterized by high-grade, rapidly progressive disease, and most commonly a serous histology ^11, 12^.

### Origin of disease

Although ovarian cancers were traditionally thought to originate from the surface epithelium of the ovary, there is strong evidence that a portion (50–60%) of high-grade serous ovarian tumors arise from the fallopian tube, and many pathologists have described pre-invasive dysplastic lesions within the distal end of the fallopian tube. These serous tubal intraepithelial carcinoma (STIC) lesions ^11, 13– 16^ often resemble high-grade serous cancer, confirming that ovarian serous cancer can originate in the fallopian tube. However, two recent studies indicate that STIC lesions sometimes represent a metastatic site rather than assumptive primary fallopian tube cancers ^17, 18^. In one study, Eckert *et al*. implanted HGSC spheroids into the fallopian tube epithelium in mice and showed that this fallopian tube growth histologically mimicked STIC lesions ^17^. In another study, McDaniel *et al*. performed targeted next-generation sequencing of an incidental STIC lesion and found that it matched the associated uterine endometrioid carcinoma, strongly indicating that the STIC lesion originated as a micrometastasis from the primary tumor ^18^. Thus, although certain aspects regarding the origins of high-grade serous ovarian cancer are understood, unanswered questions continue to challenge the field.

### Mutations and pathways

The Cancer Genome Atlas study characterized 316 primary HGSC specimens and detected *TP53* mutations in 100% of specimens and *BRCA1* and *BRCA2* mutations (both somatic and germline) in 20% ^19, 20^. Most tumors were characterized by global genomic instability. More recent advances in DNA sequencing technology have identified additional mutations in ovarian cancer, including *BARD1*, *BRIP1*, *CHEK2*, *NBN*, *PALB2*, *RAD50* family, and *NF1* ^12, 21– 26^. Given that these genes are involved in DNA repair, women with ovarian cancer, as well as their family members, are at risk of developing other cancers and thus should be referred for genetic counseling and testing ^27^.

In ovarian cancer, as in other cancers, resistance to chemotherapy is common. To identify genes conferring chemoresistance, Patch *et al*. analyzed whole genome sequences of 92 patients with HGSC to characterize mutations from three groups of tumors: those that were sensitive to platinum, those that initially responded but then developed resistance, and those that did not respond at all ^28^. They found *TP53* mutations in all samples but found only a few, low-frequency, actionable genetic alterations amongst the chemoresistant patients. These included reversion of germline mutations in *BRCA1* and *BRCA2*, mutations in the pro-apoptotic genes *FOXO1* and *BCL2L11*, and increased expression of *ABCB1*, which encodes a cellular drug efflux pump. More work is clearly needed to uncover mechanisms of chemoresistance and strategies to overcome it.

### Mechanisms of metastasis

Ovarian cancers are extremely prone to metastasize, particularly to the omentum. Two routes have been proposed to explain how ovarian cancer cells reach the omentum. First, they may travel through the bloodstream, as suggested by Sood *et al*. ^29^. These authors vascularly conjoined 15 pairs of mice, intraperitoneally injected tumor cells into “host” mice, and found that half of the conjoined “guest” mice developed omental or mesenteric metastases. Tumor cells also reached the guest mouse when the cancer cells were injected into host ovaries or vasculature. The authors further found that the metastatic cells significantly upregulated expression of epidermal growth factor receptor family genes, specifically *ErbB3*, and that targeting *ErbB3* with small interfering RNA significantly inhibited omental tumor establishment and also the size and number of tumors ^29^. A second theory is that ovarian cancer cells metastasize by shedding into the peritoneal space and then attaching to nearby structures, such as the omentum. Support for this model comes from Lengyel *et al*., who showed that omental adipocytes promote metastasis to the omentum by upregulating expression of fatty acid-binding protein 4 ( *FABP4*) ^30^. *FABP4* was strongly expressed at the adipocyte–cancer cell interface, and mice lacking *FABP4* had a significantly lower tumor burden than wild-type mice. Future work will hopefully reveal whether the hematogenous or the shedding route predominates in different ovarian cancer types so that therapies can be developed to prevent it.

### Tumor cells and the microenvironment

Mutations do not explain the full spectrum of tumor behaviors, which also depend on the tumor microenvironment, or stroma, a mixture of extracellular matrix, mesothelial cells, fibroblasts, endothelial cells, blood and lymph vessels, nerves, immune cells, and adipocytes ^31– 33^. Two hallmarks of cancer that depend on the tumor microenvironment are (1) stromal invasion and metastasis and (2) angiogenesis. Because stromal components contribute to ovarian cancer metastasis, many investigators are developing *in vitro* methods to study interactions with the tumor cells and identify strategies to inhibit metastasis by targeting tumor/microenvironment interactions. These methods include three-dimensional matrices, cancer cell spheroids, and co-cultured mesothelium (the first layer of the omentum) with cancer cells ^34^. In studies with spheroids, Davidowitz *et al*. found that tumor cell spheroids that upregulated their expression of epithelial-to-mesenchymal transition transcription factors (in particular, SNAI1, TWIST1, and ZEB1) were better able to clear the mesothelium (an essential step in metastasis) than cells that did not upregulate these factors ^35^. Additionally, receptor tyrosine kinases such as AXL and DDR2 have been found to regulate tumor cell clearance of primary, patient-derived mesothelial cells ^36, 37^. For angiogenesis, Sood *et al*. used a xenograft approach to model what happens when a patient stops taking an anti-angiogenic drug such as bevacizumab or pazopanib ^38^. They found that mice with higher circulating platelet levels had greater tumor weight and markers of proliferation and decreased levels of apoptosis. Furthermore, this group demonstrated that tumor infiltration of platelets after withdrawal of anti-angiogenic agents may contribute to rebound tumor growth. Incorporating the tumor microenvironment in future work will continue to lead to a better understanding of ovarian carcinogenesis and metastasis.

### Translational mouse models

Understanding disease pathogenesis necessitates models that mimic patient tumor behavior and interaction with the microenvironment. One approach is to develop genetically engineered mouse models (GEMMs), in which a mouse’s genome is modified to cause development of a murine disease that mimics human disease, although such models have not clearly resolved the question about the origin of ovarian tumors. Some authors have created conditional GEMMs that point to the ovary as the origin ^39– 42^, whereas other evidence points to the fallopian tube ^43, 44^. The first ovarian cancer model based on transformation of the fallopian tube epithelium as the origin was derived by using the Müllerian-specific *Ovgp1* promoter to drive expression of the SV40 large T-antigen and thus induce tumorigenesis in the fallopian tube ^45^. More recently, Perets *et al*. generated a model in which they specifically deleted *BRCA*, *TP53*, or *PTEN* in the fallopian tube and found that these mice developed HGSCs, tubal transformation, and peritoneal spread ^46^. Interestingly, when the researchers removed the ovaries, the mice developed STIC lesions and tubal transformation but not peritoneal metastasis, suggesting that the ovary plays a crucial role in the spread of IP disease ^46^.

A second type of *in vivo* model is cell line-based xenografts, in which cancer cell lines are implanted into an immunocompromised mouse or, even better, into a mouse that is syngeneic with the cell line, such as the spontaneous ovarian cancer line ID8 derived from a C57Bl/6 mouse. Although this ID8 line has been used for many years, Walton *et al*. recently sequenced it and found that it was wild-type for *TP53* and *BRCA1* and *BRCA2* ^47^, whereas 98% of human ovarian cancers contain a *TP53* mutation. Introducing a *TP53* or *BRCA2* mutation (or both) caused these cells to develop tumors and surrounding microenvironment phenotype that more closely mimicked human ovarian cancers in terms of speed and distribution of metastases.

A third approach to translational mouse models is the use of patient-derived xenografts (PDXs), which are created by implanting patient specimens into mice to study tumor behaviors. Several groups have developed ovarian cancer PDXs ^48– 51^ that respond to treatment in a manner similar to that of the patients’ tumors. For example, Landen *et al*. created subcutaneous PDX models of ovarian cancer and assessed gene expression in both the patients’ tumors and the PDXs after they developed chemoresistance ^50^. The authors identified five affected signaling pathways (protein kinase A, GNRH, sphingosine-1-phosphate, α-adrenergic, and cholecystokinin/gastrin-mediated) that were shared between the patients’ tumors and the corresponding PDXs. Models such as these provide usable platforms to study tumor and stromal elements contributing to tumorigenesis and the effects of various therapies.

## Diagnosis and prevention

### Screening in low-risk patients

No validated screening tests exist for early detection of ovarian cancer in low-risk women. Although some tests have been developed to assess known adnexal masses, the US Food and Drug Administration (FDA) recently issued a statement recommending against using any of these as screening tools in the general population ^52^. Similarly, the US Preventative Services Task Force gives screening asymptomatic women a D grade, meaning that there is moderate to high certainty that the service has no net benefit or that the harms of such a service may outweigh any benefit ^53^. For example, the FDA has approved the OVA1 test for women who have already been found to have an ovarian tumor, but it is not a screening test. The largest randomized controlled trials (RCTs) have assessed a combination of serum markers and ultrasound imaging, but these tests have proved to have inadequate sensitivity or specificity, have resulted in high rates of unnecessary interventions (65–97% of screen-positive women who underwent surgical intervention did not actually have cancer), or were unable to reduce ovarian cancer-related mortality ^54– 56^. This is an important area for future research.

### Special considerations in high-risk patients

Patients who carry particular inherited mutations warrant screening ^57^ because the lifetime risk of ovarian cancer in the general populations is 1.3% but can be as high as 40–60% in *BRCA1* and *BRCA2* mutation carriers ^58– 62^ and 10–15% in Lynch syndrome mutation carriers ^5, 63^. As additional high-risk mutations are identified in multi-gene panels, we may be able to identify more high-risk patients.

### Advances in high-risk prevention

Women who carry high-risk mutations are recommended to undergo risk-reducing bilateral salpingo-oophorectomy (RRSO) (removal of the fallopian tubes and ovaries) by age 35 to 40 for *BRCA1* and 40 to 45 for *BRCA2* ^27, 64^. A national trial (GOG-0199) comparing RRSO with longitudinal screening will hopefully clarify both the necessary screening frequency and the non-oncological outcomes of ovary removal, such as heart disease and osteoporosis ^65^, in high-risk patients.

Because removal of the ovary can cause earlier onset of menopause, increased risks to cardiac and bone health, and other impairments to quality of life, studies are underway to determine the efficacy of removing the fallopian tubes (salpingectomy) immediately but delaying ovary removal (oophorectomy) for several years. Harmsen *et al*. used previous data of cumulative ovarian cancer risk for BRCA mutation carriers to mathematically compare the risks of immediate RRSO with those of immediate salpingectomy with delayed oophorectomy ^66^. The authors concluded that a five-year delay in oophorectomy would increase the rates of ovarian cancer by 4.1% and 1.8% for those with mutations in *BRCA1* and *BRCA2*, respectively, even if the initial salpingectomy afforded no reduction of risk. Kwon *et al*. created a model to compare costs and benefits of RRSO at age 40 versus salpingectomy at age 40 with oophorectomy at age 50 in *BRCA1* and *BRCA2* mutation carriers ^67^. Although RRSO at age 40 was more effective in both cost and overall life expectancy, salpingectomy plus delayed oophorectomy resulted in higher quality-adjusted life expectancy.

### Hormonal agents in high-risk patients

An important issue to consider is that RRSO is associated with menopausal symptoms such as sexual dysfunction, hypoactive sexual desire, and less frequent sexual encounters ^68– 70^. Thus, RRSO patients may receive hormone therapy, which Kwon *et al*. assumed would not be the case in the cost–benefit analysis discussed above. Thus far, no RCTs have been conducted to address this issue. One recent systematic literature review ^71^ assessed the safety of hormone therapy in RRSO patients with BRCA mutations and found that women were likely to benefit symptomatically from hormone therapy and did not have an increased risk of breast cancer, but there was insufficient evidence regarding ovarian cancer risk. Additional studies are needed to assess ovarian cancer risk and outcomes of risk-reducing procedures in patients carrying Lynch syndrome mutations.

## Optimizing chemotherapy and developing innovative therapeutics

### Optimizing chemotherapy

Chemotherapy has long been incorporated into the care of patients with HGSC; however, the how, when, and which have become less clear as more options are available for front-line treatment. In the first decade of the 21st century, two randomized trials (GOG 114 and GOG 172) demonstrated that, after optimal tumor resection, women who received combination intravenous/IP (IV/IP) cisplatin and paclitaxel-containing chemotherapy had significantly better progression-free survival (PFS) (5.7 and 5.5 months) and overall survival (OS) (11.0 and 15.9 months) than those who received IV-only regimens ^72, 73^. This survival advantage in the IP groups was seen even though the IP patients had significant hematologic, metabolic, neurologic, and gastrointestinal toxicities, and only 71% and 42% of each study’s IP participants completed all six cycles ^72, 73^. Because of these toxicities and missed cycles, over time providers modified the regimen and instead gave patients alternate therapy schedules, reduced dosages, and substituted drugs, as described by Wright *et al*. ^74^. Another option for chemotherapy regimen was provided by a Japanese study showing that a lower but more frequent dose of paclitaxel (“dose-dense paclitaxel”) led to improved PFS and OS ^75^. Additionally, the GOG-0218 trial demonstrated that the use of bevacizumab in the front-line and maintenance setting improved PFS by 3.8 months when compared with conventional every-3-weeks carboplatin and paclitaxel ^76^.

Recently, the GOG-0252 trial was undertaken in an attempt to identify the best front-line regimen given the improved survival data seen with IP chemotherapy and dose-dense paclitaxel when compared with conventional every-3-weeks carboplatin and paclitaxel. This study had three arms, each of which included bevacizumab therapy in addition to (1) IV dose-dense paclitaxel and IV carboplatin, (2) IV dose-dense paclitaxel and IP carboplatin, and (3) IV/IP paclitaxel with IP cisplatin at a reduced dose. PFS and toxicities were found to be similar amongst all three treatment regimens, although some participant cross-over between arms may have clouded results ^77^. We are awaiting OS data as they mature to help determine whether there is a superior front-line regimen.

Not only has the best route been intensely debated but the optimal timing of therapy has been and is currently being studied. Chemotherapy is usually given either (1) only after primary debulking surgery (PDS) or (2) as both neoadjuvant chemotherapy (NACT) before and after interval debulking surgery (IDS). The goal of any cytoreductive surgery is to maximally reduce the disease, as doing so is well known to improve patient outcomes ^78– 80^. However, recent trials have tried to determine whether patients receiving NACT and post-IDS chemotherapy have better outcomes than those receiving only chemotherapy after PDS. Initially, several large RCTs found that the NACT/IDS regimen was non-inferior to the PDS regimen in terms of PFS and OS and overall morbidities. However, the OS of the groups was lower than expected, suggesting that the included patients somehow differed from the larger collective population of patients with HGSC ^81– 84^. Subsequent reports noted that recurrences after NACT/IDS regimens were more likely to be platinum-resistant and were less responsive to second-line therapies than those that occurred after PDS regimens, and some studies even demonstrated better PFS and OS in those who received regimens after PDS ^85– 90^ when compared with those after NACT/IDS. There is likely a contributing biological factor, yet to be determined, for tumors that present as unresectable versus those that can be resected to no residual disease with PDS. Controversy with NACT/IDS exists given the possibility of a compromised responsiveness to additional lines of therapy including platinum-containing agents and possibly a risk of higher recurrence rates. Nonetheless, a NACT/IDS regimen may be the best option for patients with particular comorbidities, histological subtypes, or other clinical situations making them unable to tolerate an aggressive, up-front surgical procedure.

### Developing innovative therapeutics

Traditionally, ovarian cancer has been treated with cytotoxic agent regimens chosen on the basis of cancer stage, and most patients have eventually developed chemotherapy resistance, leading to overwhelming disease burden and death. Researchers are working to find innovative methods to restore chemosensitivity and develop adjuvants to improve the function of cytotoxics to decrease required doses and improve toxicity profiles. In addition, researchers are investigating novel agents targeting specific tumor mutations. We highlight some promising findings below.

### Preclinical development

Nanoparticle technology is a promising method by which to introduce therapeutics and minimize off-target effects. In one study, Landen *et al*. treated a mouse model of ovarian cancer with nanoliposomal particles containing small interfering RNAs targeting cancer stem cells in combination with either docetaxel or cisplatin ^91^. This regimen reduced tumor growth more than chemotherapy alone. In another study, researchers used nanoliposomal particles to target a cell membrane transporter involved in cellular extrusion of chemotherapeutic drugs. This approach was able to restore paclitaxel sensitivity both *in vitro* and *in vivo* ^92^. A nanoparticle derived of a naturally occurring alcohol was recently demonstrated to improve apoptosis and inhibit tumor growth when combined with paclitaxel *in vitro* and *in vivo* ^93^. Other promising therapeutics target various parts of the surrounding tumor stroma. For example, Wang *et al*. found that the peptide prosaposin could inhibit metastasis in a platinum-resistant PDX model by stimulating the release of anti-tumorigenic protein thrombospondin-1 from surrounding monocytes ^94^.

### SHIVA and MATCH clinical trials

Given the success of targeted therapies based on tumor mutational status in lung cancer and melanoma, this approach is being investigated in other solid cancers, including ovarian cancer. The SHIVA (A Randomized Phase II Trial Comparing Therapy Based on Tumor Molecular Profiling Versus Conventional Therapy in Patients With Refractory Cancer) trial in France was a multicenter, phase II RCT of 195 histology-agnostic, heavily pretreated patients (of whom 29 had ovarian cancer). Patients were randomly assigned to either the physician’s choice of drug or a molecularly targeted agent that was matched to the patient tumor molecular profile but was not approved for that tumor type. Although the study reported no difference in PFS between the two groups ^95^, the study was powered to detect only a 15–30% improvement and may have missed smaller effects. In the ongoing National Cancer Institute-initiated Molecular Analysis for Therapy Choice (MATCH) trial, all recurrent, solid tumors undergo targeted exome sequencing to identify mutations, and the patients are treated with a matched targeted therapy until disease progression ^96^. This ongoing trial includes patients with ovarian cancer and will provide useful information about the value of this genomics-based approach to treatment.

### FDA-approved agents

Only two targeted agents are currently FDA-approved for use in ovarian cancer. Olaparib, a poly-ribose polymerase (PARP) inhibitor, was approved in 2014 for use in patients with *BRCA1/2* mutations who have been treated with three or more previous lines of chemotherapy. Bevacizumab, a vascular endothelial growth factor (VEGF) anti-angiogenic, also received FDA approval in 2014 for use in recurrent, platinum-resistant patients in combination with paclitaxel, topotecan, or pegylated liposomal doxorubicin. A number of other targeted therapeutics, including additional PARP inhibitors, anti-angiogenics, tyrosine kinase inhibitors, and immunotherapeutics, are currently being investigated ( Table 1).

**Table 1.** Recent and ongoing clinical trials using targeted therapeutics for high-grade serous Müllerian adenocarcinomas.

Class	Agent	Target(s)	Trial group/name, phase	Progression-free survival and overall survival, months
Anti-angiogenics				
	Bevacizumab ^a^	VEGF	ICON-7, phase III ^101^ GOG-0218, phase III ^76^ OCEANS, phase III ^102^ AURELIAa, phase III ^103^	PFS 19.8/OS 36.6 PFS 11.2+14.1/OS 38.7+39.7 PFS 12.4/OS 33.3 PFS 6.7/OS 16.6
	Cabozantinib	c-met, VEGFR2, RET, AXL	*NRG-GY001, phase II* ^104^	-
	Cediranib	VEGFR2/3/4, c-kit	Multicenter, phase II ^105^ *NRG-GY004, phase III* ^106^ *NRG-GY005, phase II/III* ^107^	PFS 5.2/OS 16.3 - -
	Fosbretabulin	Endothelial microtubules	GOG-0186I, phase II ^108^	PFS 7.3/OS 24.6
	Trebananib/AMG386	Angiopoietin-1/2	TRINOVA1, phase III ^109, 110^ *TRINOVA2-3, phase III* ^111, 112^	PFS 7.2/OS 19.3 -
PARP inhibitors				
	Olaparib ^a^	PARP	Multicenter ^a^, phase II ^113^ Multicenter, phase II ^114, 115^ *NRG-GY004, phase III* ^106^ *NRG-GY005, phase II/III* ^107^	PFS 7.0/OS 16.6 PFS 8.4/OS 29.8 ^b^ - -
	Niraparib	PARP	ENGOT-OV16/Nova Trial (multi-arm), phase III ^116^ *QUADRA, phase II* ^117^	PFS 21+12.9+9.3 /OS not yet mature -
	Veliparib	PARP	*GOG-3005, phase III* ^118^	-
	Rucaparib	PARP	*ARIEL2-4, phase II/III* ^119– 121^	-
	Pazopanib	PARP	*GOG-0186J, phase II* ^122^	-
	Cabozantinib	PARP	*GOG-0186K, phase II* ^123^	-
Immunologics				
	EGEN-001	IL-12	GOG-0170Q, phase II ^124^	PFS 2.9/OS 9.2
	Nivolumab	PD-1	*NRG-GY003, phase II* ^125^	-
	Ruxolitinib	JAK1, JAK2	*NRG-GY007, phase I/II* ^126^	-
	VTX-2337	TLR8	*GOG-3003, phase II* ^127^	-
Pathway inhibitors				
	Temsirolimus	MTOR	GOG-0268, phase II ^128^	PFS 3.2/OS 11.6

^a^US Food and Drug Administration approval gained. ^b^Overall survival (OS) may be underestimated; OS = 31.9 months when crossover poly-ribose polymerase (PARP) exposure post-trial is included ^129^. Italics indicate trial in progress or awaiting data analysis. GOG, Gynecologic Oncology Group; IL-12, interleukin-12; JAK, Janus Kinase; MTOR, mammalian target of rapamycin; NRG, NSABP/RTOG/GOG Collaborative Group; PD-1, programmed death 1; PFS, progression-free survival; TLR8, Toll-like receptor 8; VEGF, vascular endothelial growth factor; VEGFR2, vascular endothelial growth factor receptor 2.

### Immunotherapy

The possible effectiveness of immunotherapeutic approaches in ovarian cancer was suggested by a 2003 study reporting that ovarian cancer patients whose tumors contained CD3 ^+^ T cells experienced a higher five-year OS rate than those whose tumors did not contain those cells (38% versus 4.5%). In another approach, patients are “immunized” with tumor antigens. Although positive antibody responses have been reported, no vaccination approach has yet improved any clinically relevant outcomes in ovarian cancer ^97– 99^.

In several cancer types, investigators are attempting to block programmed death 1 (PD-1), a protein that protects tumor cells from immune system attack, but so far little work has been done in this area for ovarian cancer. In a single phase II study, the anti-PD-1 antibody nivolumab produced an overall response rate of 15% (3 out of 20) and a median PFS of 3.5 months. Similarly, in a preliminary report of a phase IB study using the anti-PD-1 antibody pembrolizumab in patients with ovarian cancer that expressed the PD-1 ligand, the overall response rate was 3 out of 20 ^100^. Although the overall response rate was low, two patients had complete and durable remissions of up to one year.

### Conclusions

Since the incorporation of taxane-containing chemotherapy into standard treatment, the survival of patients with ovarian cancer has improved only slightly. However, the current research highlighted here gives us hope that survival rates will continue to increase as there is further development of *in vivo* mouse models, *in vitro* tumor microenvironment models, identification of pathways activated in chemoresistance, immunotherapy, optimization of chemotherapy regimens, and development of targeted agents. In addition, cost models are needed to determine the feasibility and sustainability of widespread usage of newly developed approaches.
